# Enhanced CT combined with texture analysis for differential diagnosis of pleomorphic adenoma and adenolymphoma

**DOI:** 10.1186/s12880-023-01129-9

**Published:** 2023-10-27

**Authors:** Feifei Xia, Foqing Guo, Zhe Liu, Jie Zeng, Xuehua Ma, Chongqing Yu, Changxue Li

**Affiliations:** Department of Oral and Maxillofacial Surgery, the First Affiliated Hospital of Shihezi University, Shihezi, 832000 China

**Keywords:** Enhancement CT, Texture analysis, Pleomorphic adenoma (PA), Adenolymphoma (AL)

## Abstract

**Objective:**

This study sought to evaluate the worth of the general characteristics of enhanced CT images and the histogram parameters of each stage in distinguishing pleomorphic adenoma (PA) and adenolymphoma (AL).

**Methods:**

The imaging features and histogram parameters of preoperative enhanced CT images in 20 patients with PA and 29 patients with AL were analyzed. Tumor morphology and histogram parameters of PA and AL were compared. Area under the curve (AUC), sensitivity, and subject operational feature specificity (ROC) analysis were used to determine the differential diagnostic effect of single-stage or multi-stage parameter combinations.

**Results:**

The difference in CT value and net enhancement value of arterial phase (AP) were significant (p < 0.05); Flat sweep phase (FSP), AP mean, percentiles, 10th, 50th, 90th, 99th and arterial period variance and venous phase (VP) kurtosis in the nine histogram parameters of each period (p < 0.05). An analysis of the ROC curve revealed a maximum area beneath the curve (AUC) in the 90th percentile of FSP for a single-parameter differential diagnosis to be 0.870. The diagnostic efficacy of the mean value of FSP + The 90th percentile of AP + Kurtosis of VP was the best in multi-parameter combination diagnosis, with an AUC of 0.925, and the sensitivity and specificity of 0.900 and 0.850, respectively.

**Conclusion:**

The histogram analysis of enhanced CT images is valuable for the differentiation of PA and AL. Moreover, the combination of single-stage parameters or multi-stage parameters can improve the differential diagnosis efficiency.

## Introduction

Among the three major salivary glands in the human body, the parotid gland, as the largest salivary gland, accounts for about 3% of head and neck tumors, most of which are benign tumors about 80%, and pleomorphic adenoma (PA) and adenolymphoma (AL) are the two most common benign tumors, accounting for 80% and 10% [1–3], respectively. Although both PA and AL were benign tumors, there were large differences in their biological behavior, surgical methods, and postoperative outcome. About 2 -25% of PA is malignant, If the tumor is removed only along the capsule, about 15% of cases will relapse, so PA only needs to retain the facial nerve during the operation, remove the tumor and the involved parotid gland together. However, AL rarely has malignant changes, and most of the capsule is complete, the tumor only needs to be removed along the capsule. Unfortunately both PA and AL exhibit similar clinical characteristics, making it difficult to distinguish them. Therefore, accurate preoperative differential diagnosis is very important for the perioperative preparation, condition communication and the selection of surgical methods [4–7].

At present, there are many methods for diagnosing and differentiating salivary gland tumors, for example, by radiological tests such as computed tomography (CT), ultrasonography (US), and magnetic resonance imaging (MR). Among them, the US examination for salivary gland tumors has many advantages, including low cost, ease of operation, and high safety, but for tumors located in the deep lobes, the test results are not very ideal, and the quality of the test results is closely related to the personal level of the physician and his medical experience, and different physicians may obtain different results [8]. MR is indicated for non-invasive determination of different histological subtypes of parotid gland tumors. However, it is not indicated in patients with metal prostheses and pacemakers, which may affect the diagnosis, be expensive, and take longer [9]. Currently, CT is not the preferred test for the evaluation of parotid gland tumors, however, CT has high diagnostic value for assessing the extent of tumors, especially those located in deep regions, and can help clinicians evaluate tumors more accurately, and CT can also detect lymphadenopathy to help identify benign or malignant tumors for appropriate treatment [10]. Contrast contrast-enhanced CT can clearly observe the relationship between the lesion and surrounding tissues, organs, and blood vessels, and although there are reports that contrast may cause adverse effects, the overall prevalence of adverse reactions is about 0.7%, of which more than 80% are mild [11]. In the past, there were few studies on enhanced CT imaging, and the results were not very satisfactory, and no diagnostic system has been formed, and CT imaging focuses on the morphological characteristics of tumor margin, size, location, and density [12, 13]. In addition, physicians evaluate CT results based on their own skills and clinical experience, so that their results are more biased towards supervisor’s judgment than objective indicators.

The histogram analysis method provides more and more detailed quantitative information on image heterogeneity by quantifying the grayscale information of lesion images, calculating the characteristic parameters of ROI in the image, evaluating the relationship and distribution of ROI gray intensity, and providing more detailed quantitative information on image heterogeneity, which has the advantages of simple operation process, objective data quantification and strong reproducibility of results [14, 15]. So far, many research results have shown that histogram analysis has high medical value for diagnosing benign and malignant tumors, evaluating treatment effects, and predicting prognosis [16–18]. In this study, the imaging features and the histogram parameters of PA and AL were analyzed to explore the feasibility and value of enhanced CT lesion imaging features and histogram analysis in identifying PA and AL.

## Materials and methods

### Patient selection

**Fig. 1:**
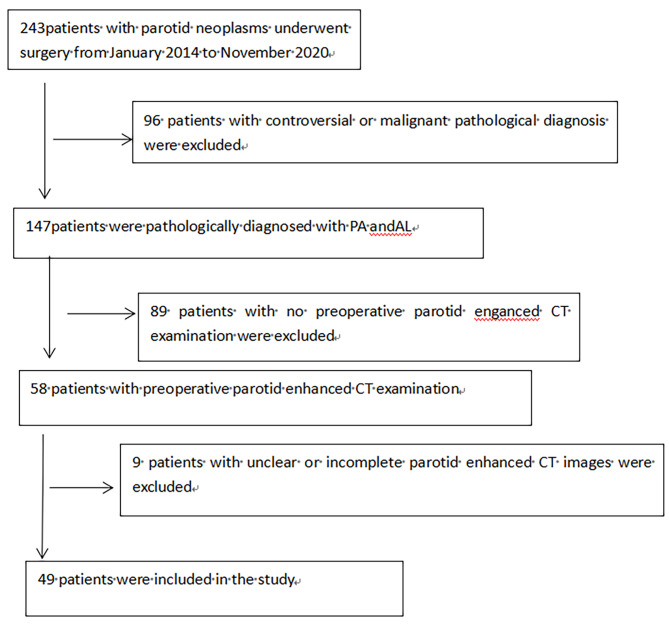
The recruitment pathway of patients in this study

This study collected 243 patients with parotid gland tumors who visited the First Affiliated Hospital of Shihezi University School of Medicine from January 2014 to November 2020. Each patient was carefully screened to exclude 96 patients (tumours malignant or controversial), and the remaining patients were diagnosed with PA or parotid AL and met the inclusion criteria. Each patient was screened for preoperative parotid gland contrast CT, and 89 patients without preoperative contrast CT were excluded. The remaining patients with contrast-enhanced CT were analyzed and nine patients were excluded due to unclear contrast-enhanced CT images. In the end, 49 patients remained. A total of 49 patients were included in this study (Fig. 1). Patients who met the inclusion criteria were divided into two groups, 20 in the PA group (11 males and 9 females) and 29 in the AL group (27 males and 2 females). The Ethics Committee of the First Affiliated Hospital of Shihezi University School of Medicine approved the study. As the study was retrospective and non-interventional, written informed consent was waived.

### Enhanced CT examination

A Philips Brilliance 16 row CT scan (Discovery CT750 HD, GE Medical Systems, Waukesha, WI, USA) ranged from the external ear foramen to the lower margin of the mandible. Scanning parameters: 200mAs, 120KV, matrix 512, the reconstruction layer thickness of 1 mm. Conventional flat sweep phase (FSP) images were acquired before performing enhanced CT examinations. doxitol (1.5ml/Kg, Iopamidol, Jiang Su Heng Rui Pharmaceutical, Jiangsu, China) was injected through the antecubital vein at a flow rate of 2.5 ml/s, and 25ml of saline at the same flow rate, arterial phase(AP) and venous phase(VP) scans after 30-35s and 55- 60s delay, respectively.

### Image acquisition and analysis

The common characteristics of parotid gland lesions during FSP were analyzed by the chief physician of oral and maxillofacial surgery and experienced physicians, jointly, and it was ensured that neither physician looked at the pathological diagnosis of the disease during the analysis. The following FSP CT image features of tumor morphology in the 49 lesions were analyzed: (1) Boundary, classified as clear (with complete capsule echo) or unclear (blurred with surrounding parotid tissue, no obvious capsule echo). (2) Shape, classified as regular (parotid gland tumor lesions clearly demarcated from surrounding normal parotid tissue) or irregular (parotid tumor lesions with blurred demarcation from surrounding parotid tissue). (3) The homogeneity of the focal area, characterized as homogeneous (uniform grayscale within parotid tumor lesions) or heterogeneous(uneven grayscale within parotid tumor lesions with high- or low-density imaging). (4) Calcifications, classified as (high-density shadow) or absence (low-density shadow). They jointly measure the CT value of the tumor parenchyma area, and calculate the double-stage CT net enhancement value (net enhancement value = enhancement CT value - FSP CT value).

**Fig. 2:**
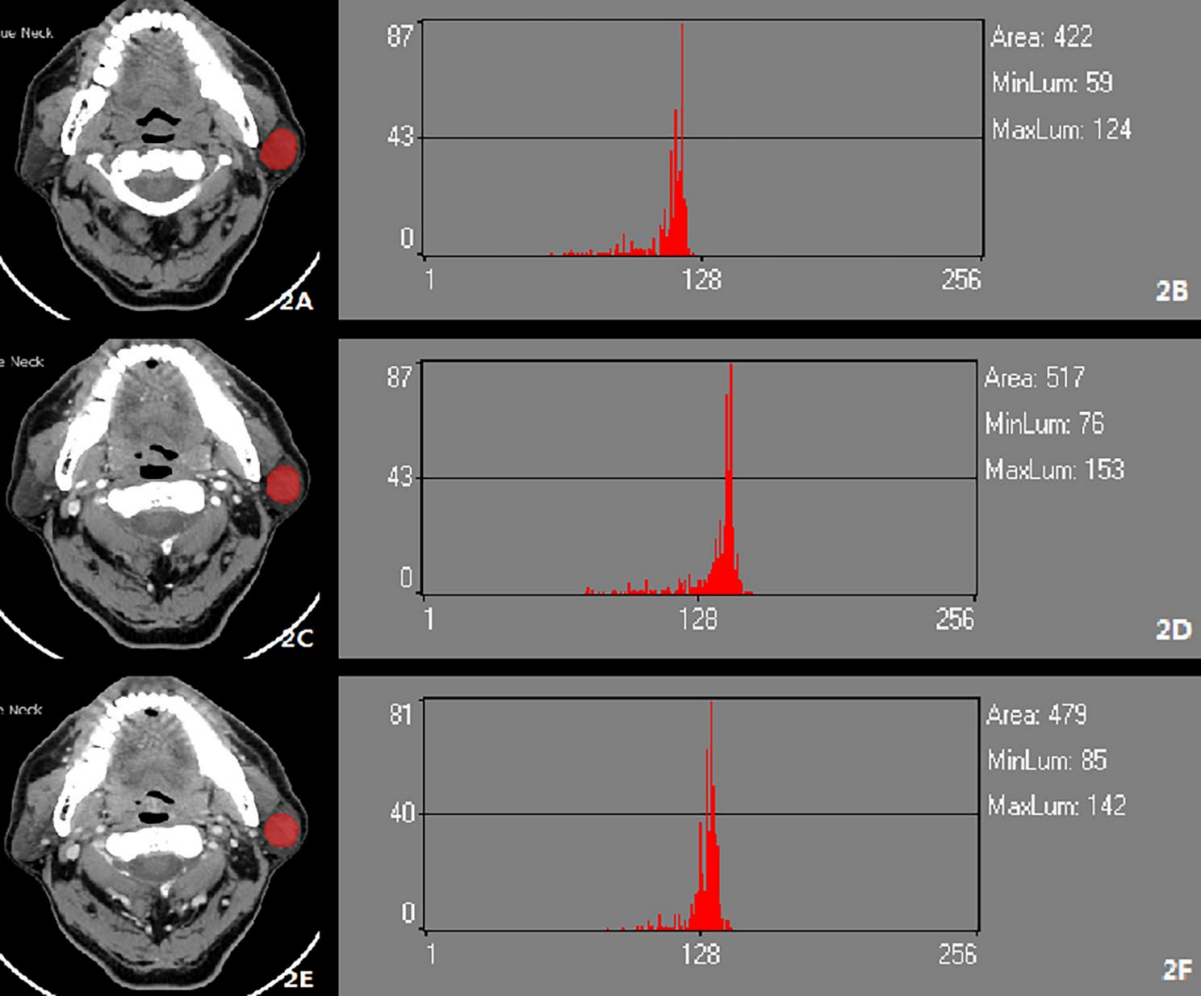
Left pleomorphic adenoma (**A**) ROI in FSP of Enhanced CT; (**B**) The histogram of ROI in FSP of Enhanced CT; (**C**) ROI in AP of Enhanced CT; (**D**) The histogram of ROI in AP of Enhanced CT; (**E**) ROI in P of Enhanced CT ; (**F**) The histogram of ROI in VP of Enhanced CT

**Fig. 3:**
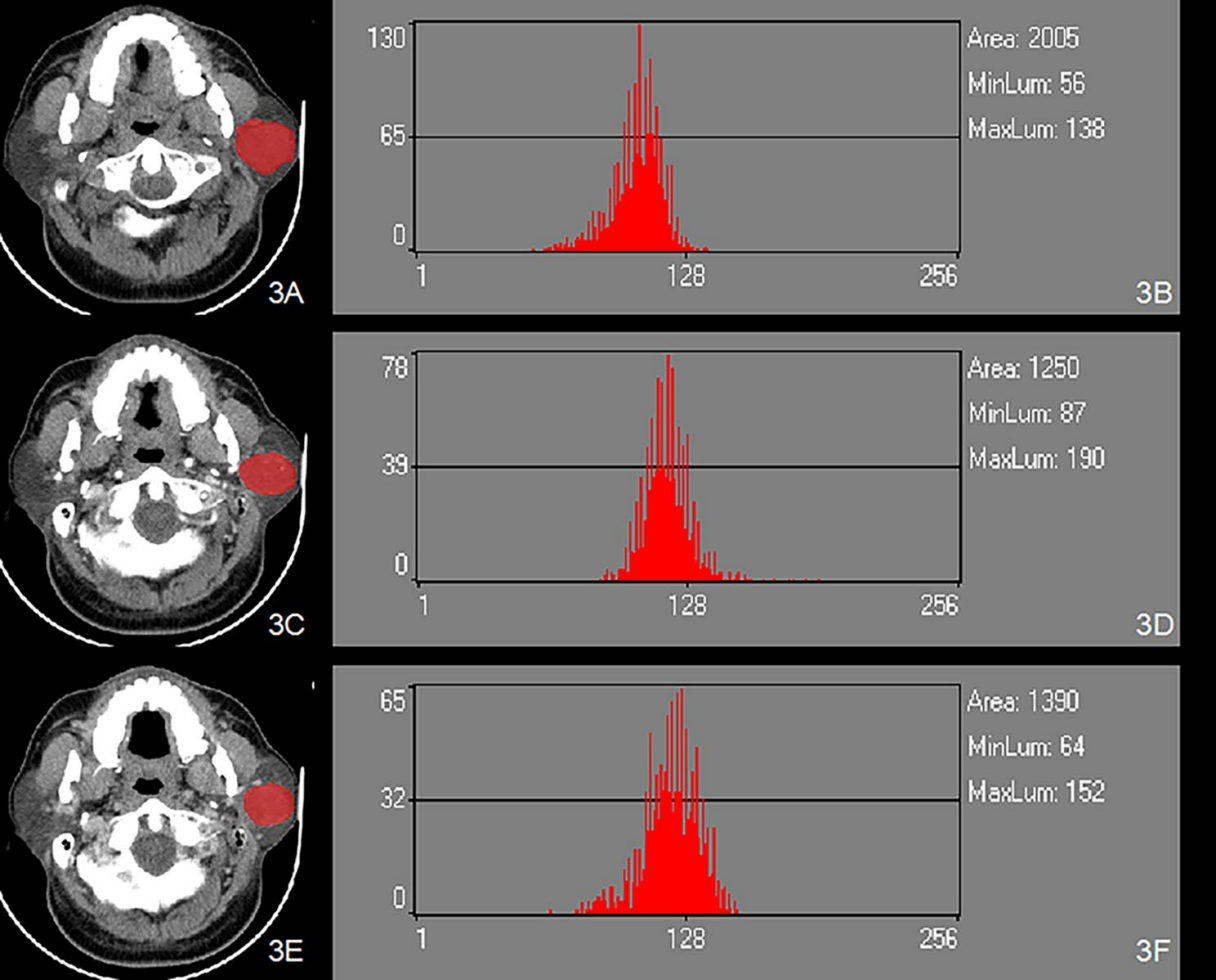
Left adenolymphoma (**A**) ROI in FSP of Enhanced CT; (**B**) The histogram of ROI in FSP of Enhanced CT; (**C**) ROI in AP of Enhanced CT; (**D**) The histogram of ROI in AP of Enhanced CT; (**E**) ROI in P of Enhanced CT ; (**F**) The histogram of ROI in VP of Enhanced CT

After downloading the images through the picture archiving and communication systems (PACS) and selecting transaxial CT images of the largest level of the tumor in each case, a physician well-experienced in CT diagnosis and a chief physician in oral and maxillofacial surgery who both physicians were blinded to the pathological diagnoses of the tumors used Mazda4.6 software (http://www.eletel.p.lodz.pl/mzada/) [19] together to complete the histogram texture analysis. Before texture feature extraction, all images were standardized their gray scale level in the range of [µ-3δ, µ + 3δ](µ andδare mean gray value and standard deviation, respectively) to reduce the influence of contrast and brightness changes. Duo to analyse histograms, region of interest (ROI) was drawn along the edge of the parotid tumors and a consensus was reached on the most appropriate ROI for each lesion by 2 fore-mentioned physicians. Histogram data were automatically generated by used Mazda for each ROI, and the following parameters were derived: mean, variance, skewness, kurtosis, and the 1st, 10th, 50th, 90th, and 99th percentiles (perc1st, 10th, 50th, 90th and 90th). Mean was defined as the average value of pixel intensity (i.e., echogenicity on gray scale ultrasound) ranging from 1 (black on gray scale ultrasound) to 256 (white on gray scale ultrasound images). variance was the variance of pixel intensity. Skewness was a scale of asymmetry based on the mean. (The skewness for the symmetric distribution is zero. Negative values of skewness indicate data that are skewed left, meaning that the left tail is long relative to the right tail.) Kurtosis was peakedness indicative of whether the histogram distribution was concentrated into an average value. (Positive kurtosis indicates a peaked distribution, and negative kurtosis indicates a flat distribution). The parameters of perc1st, 10th, 50th, 90th and 90th were derived for the cumulative histogram (The nth percentile was the point at which n% of the voxel values that form the histogram were found to the left), [20] as shown in Figs. 2 and 3.

### Statistical analysis

Using SPSS 22.0 (SPSS Inc., Chicago, IL, USA), a statistical analysis of the data was conducted. A chi-square test was employed to quantify the count data (Employ the Fisher exact probability method or the continuous correction method that does not meet the requirements of the chi-square test), To assess normality and homogeneity of variance, numerical data is employed, if all are met, an independent-sample t-test and a nonparametric rank sum test are performed. The chi-square test was used to compare the difference of boundary, shape, homogeneity, and Calcification among PA and AL. Due to the small numbers of expected frequencies in some cells, the Continuity Correction was used to compare the difference of homogeneity and Calcification among PA and AL. GraphPad Prism7.0 (San Diego,California USA) was used to plot the ROC curves of single statistically significant histogram parameters and pairwise joint model, with the sensitivity as the ordinate and 1-specificity as the abscissa [21, 22]. The AUC was calculated, and then analyzing its diagnostic effect. P-values less than 0.05 are considered statistically significant.

## Results

### Comparison of Tumor morphology and CT values for each phase

The boundary, shape, homogeneity, and calcification of the PA and AL lesions were not significantly different between the two groups (p > 0.05). Table 1 demonstrated that the AP CT and net reinforcement values of the AL group were superior to those of the PA group, and the statistical significance was significant (p < 0.05).

**Table 1:** Comparison of tumor morphology and CT values for each phase

Parameters		PA	AL	*F/X* ^*2*^	*Pairwise*
Boundary	Clear	14	22	0.090	0.648
	Unclear	6	7		
Shape	Regular	8	11	0.021	0.884
	Irregular	12	18		
homogeneity	Homogeneous	17	20	1.646	0.200
	Heterogeneous	3	9		
Calcification	Yes	3	4	0.014	0.609
	No	17	25		
Mean CT value of FSP		26.85 ± 12.41	41.58 ± 11.45	0.102	0.751
Mean CT value of AP		42.50 ± 18.89	81.38 ± 31.89	5.106	0.029*
Mean CT value of VP		50.25 ± 17.25	67.24 ± 21.23	0.217	0.644
Net reinforcement value of AP		15.65 ± 13.00	39.79 ± 25.84	13.894	0.001*
Net reinforcement value of VP		23.40 ± 14.13	25.66 ± 14.62	0.049	0.825

*, Statistically significant difference. *P* < 0.05 for pairwise comparisons

AL, adenolymphoma; PA, pleomorphic adenoma; FSP, flat sweep phase; AP, arterial phases; VP, venous phases

### Comparison of histogram parameters for each phase

**Fig. 4:**
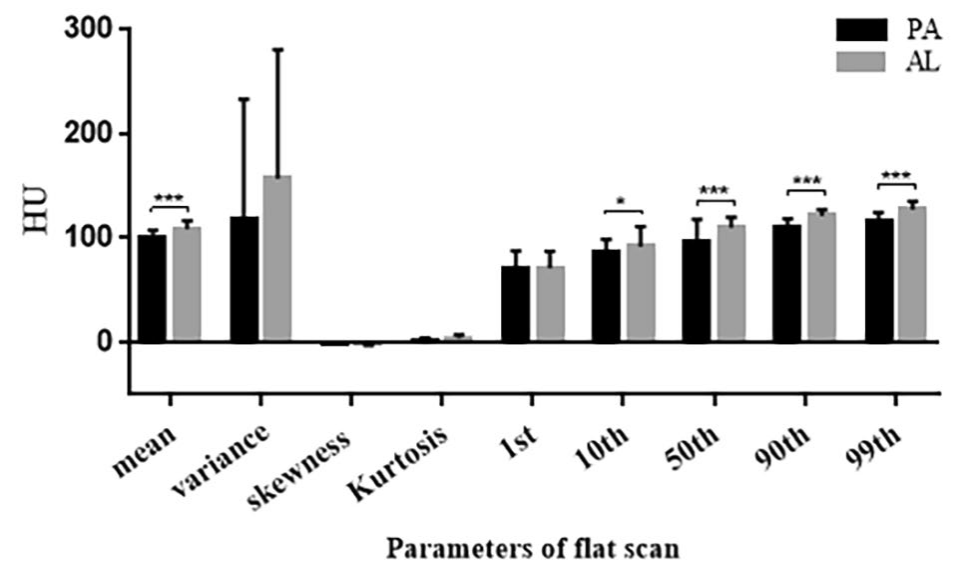
Histogram comparison of histogram parameters for Enhanced CT FSP between PA and AL groups

**Fig. 5:**
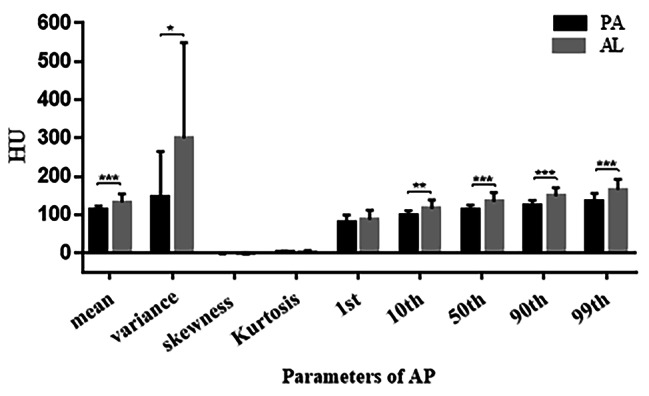
Histogram comparison of histogram parameters for Enhanced CT AP between PA and AL groups

**Fig. 6:**
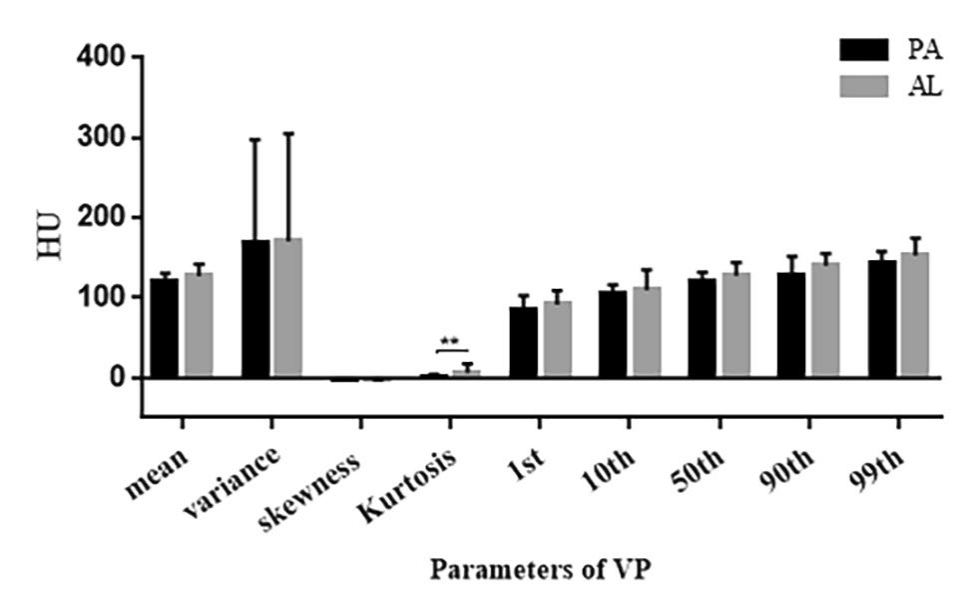
Histogram comparison of histogram parameters for Enhanced CT VP between PA and AL groups

The mean, 10th, 50th, 90th, and 99th percentiles of the AL group in FSP were all greater than those of the PA group, with statistically significant differences (Fig. 4).The mean, variance, 10th, 50th, 90th and 99th percentiles of the AL group in AP were greater than the PA group, with statistically significant differences (Fig. 5).In the VP, only the kurtosis values in group AL was greater than that in the PA group, and none of the remaining parameters were statistically significant (Fig. 6).

### ROC analysis

The AUC of the mean CT value of AP was 0.795. The AUC of the AP net reinforcement value was 0.787. The maximum AUC of 90th in the FSP parameter was 0.869, and when the cutoff was 118.504, the sensitivity, specificity, and Jordan index were 0.704, 0.904, and 0.602, respectively. The maximum AUC of 90th in the AP parameters was 0.838, and when the cut-off value was 135.505, the sensitivity, specificity, youden index were 0.774, 0.804, 0.577, respectively. Only the kurtosis values in the VP was statistically significant between the two groups, with an AUC of 0.710, and when the cut-off value was 1.710, the sensitivity, specificity, youden index were 0.735, 0.654, 0.384, respectively (Table 2). The multi-parameter combined diagnostic ROC curve showed the best diagnostic efficacy of Mean of FSP + The 90th percentile of AP + Kurtosis of VP with an AUC of 0.937 and a sensitivity and specificity of 0.902 and 0.851, respectively (Table 3).

**Table 2:** ROC analysis of solitary parameters to diagnose PA, and AL

Parameters	AUC(95%CI)	sensitivity	specificity	Youden index	Optimal cutoff value	*P*
Mean CT value of AP	0.795(0.673–0.917)	0.671	0.954	0.623	71.501	0.000
Net reinforcement value of AP	0.787(0.663–0.910)	0.632	0.852	0.483	28.504	0.001
The mean of FSP	0.832(0.713–0.951)	0.670	0.954	0.626	108.520	0.000
The 10th percentile of FSP	0.694(0.546–0.842)	0.634	0.752	0.383	94.504	0.021
The 50th percentile of FSP	0.820(0.693–0.947)	0.836	0.802	0.634	106.506	0.000
The 90th percentile of FSP	0.869(0.776–0.972)	0.704	0.904	0.602	118.504	0.000
The 99th percentile of FSP	0.838(0.725–0.951)	0.933	0.603	0.532	118.507	0.000
The value of mean of AP	0.795(0.673–0.917)	0.706	0.807	0.504	117.054	0.000
Variance of AP	0.695(0.540–0.850)	0.973	0.455	0.423	57.580	0.021
The 10th percentile of AP	0.747(0.610–0.883)	0.671	0.858	0.524	105.507	0.003
The 50th percentile of AP	0.779(0.652–0.907)	0.532	0.955	0.486	133.502	0.001
The 90th percentile of AP	0.828(0.714–0.941)	0.774	0.804	0.577	135.505	0.000
The 99th percentile of AP	0.786(0.662–0.909)	0.601	0.857	0.152	153.506	0.001
Kurtosis of VP	0.710(0.565–0.855)	0.735	0.654	0.384	1.710	0.013

FSP, flat sweep phase; AP, arterial phases; VP, venous phases

**Table 3:** ROC analysis of multiple parameters to diagnose PA, and AL

Parameters	AUC(95%CI)	sensitivity	specificity	Youden index	Optimal cutoff value	*P*
The mean of FSP + The 90th percentile of FSP + The 99th percentile of FSP	0.867(0.768–0.965)	0.930	0.703	0.630	0.473	0.000
The mean of AP + The 90th percentile of AP + The 99th percentile of AP	0.843(0.780–0.943)	0.808	0.807	0.606	0.602	0.000
The mean of FSP + The 90th percentile of AP + Kurtosis of VP	0.937(0.873-1.000)	0.902	0.851	0.750	0.542	0.000

FSP, flat sweep phase; AP, arterial phases; VP, venous phases

## Discussion

The most prevalent benign parotid gland tumor, PA, is more prevalent in females than males, with a more painless growth rate and a prolonged illness. 15% of cases had a postoperative risk of recurrence, with uniform or uneven CT plain density, and well-demarcated [23–25]. AL, also known as Voxinoma, is more common in middle-aged and elderly men with smoking. The risk of malignant change is less than 1% and rarely postoperative recurrence. Its CT plain scan is less uniform and slightly dense and with clear edge [26–28]. In this study, the general characteristics of the two lesion images and the CT values of each period were compared, and we found that only the CT values of AP and the net enhancement value of AP were statistically different between the two groups, and both had certain differential diagnostic efficacy, which was basically consistent with the research conclusion of Seung Hoon Woo et al [26].

At present, the differentiation of PA and AL is mainly through the above imaging features, which is the key diagnostic mode, however, based on the analysis of physicians is subjective and often affected by many factors, such as the size of the lesion, the clarity of the image, and the physician’s personal ability and experience, this study based on the average CT value and AP net enhancement value to distinguish the AUC of PA and AL is 0.79 and 0.78, respectively. The accuracy of traditional imaging features still needs to be improved to distinguish between the two.

Texture analysis is different from the competent image feature analysis, which quantifies the grayscale information of the image, reflects the microstructure and internal biological indicators of tumor tissue with objective indicators, provides more tumor heterogeneity information that cannot be observed by the naked eye, and distinguishes malignant tumors from normal tissues or benign lesions mainly through tumor heterogeneity [14, 15]. Histogram analysis is a useful texture analysis method that the main parameters include the mean, variance, skewness, scale and the 1st, 10th, 50th, 90th and 99th percentiles [19]. The parameters of the histogram can reflect the structure and heterogeneity of tumor tissue to a certain extent, and the average value can reflect the central trend and average level of the data. The variance is used to describe the degree of dispersion of gray values, that is, it can reflect the heterogeneity of the lesion components, the larger the variance, the more the data deviate from the mean, the stronger the heterogeneity; The skewness value is an indicator to measure the asymmetry of the gray value, the larger the absolute value of the skewness, the more asymmetric the distribution; The kurtosis value indicates that compared with the normal distribution, it can reflect the relative steepness of the distribution, the smaller the kurtosis value, the flatter the gray value distribution, and the more complex the tumor tissue components. The percentile reflects the structure of the grayscale histogram and quantifies the intra-tumor heterogeneity [29, 30]. This study is based on CT texture analysis, and aims to investigate the value of the general features of enhanced CT images and the histogram parameters of each stage to identify PA and AL.

In the current study, the enhanced CT data of 20 PAs and 29 ALs were retrospectively analyzed, obtained the histogram characteristic parameters of the largest ROI of the tumor lesion, and found statistical differences in the mean, 10th, 50th, 90th, 99th percentile of AP and FSP and variance of AP and kurtosis of VP. We found that the mean, 10th, 50th, 90th, 99th percentile of AL was higher than the PA during FSP and the different parameters increase the variance during AP. We speculate that the histologic differences between the two kinds of tumors were reflected on these histogram parameters differences. As the name implies, PA is also known as mixed tumor, and the tumor composition is often composed of a mixture of two different germ layer tissues, with a rich and diverse tissue structure, the basic structure includes glandular epithelium, myoepithelium, mucus, mucus tissue [31]. Low HU on early CT images of PA may be due to the abundance of mucus-like or fibrous matrix elements and low microvascular density, which is largely consistent with earlier findings [20, 23, 25]. In contrast, high HU on early WT CT images may be due to histological features consisting of epithelial, simple, predominantly cancer cell components, and lymphoma stroma with a higher internal microvascular density [24, 26–28]. In addition, the study also found that only kurtosis showed a difference during VP. We tried to explain this conclusion using its histopathological features and vascular structure [32]. Histopathology, ALs have a high microvascular count and high cellularity, in contrast, PAs are with a large amount of mucinous matrix and rare epithelial components. Characteristic dynamic CT of ALs presents with rapid contrast enhancement at AP, decreased enhancement at VP, and shows slow flushing and shows steady horizontal enhancement, while PA gradually increases, which we speculate may be due to slow leakage of contrast medium from a small amount of microvascular into the vascular space and mucus-like matrix.

ROC analyses were performed for the ability of these parameters with statistically significant differences, individually and combined, to discriminate between PA and AL. The AUC of the mean CT value of AP was 0.79. The AUC of the AP net reinforcement value was 0.78. The maximum AUC of 90th in the FSP parameter was 0.87. The maximum AUC of 90th in the AP parameters was 0.83, and only the kurtosis values in the VP was statistically significant between the two groups, with an AUC of 0.71. The multi-parameter combined diagnostic ROC curve showed the best diagnostic efficacy of Mean of FSP + The 90th percentile of AP + Kurtosis of VP with an AUC of 0.93 and a sensitivity and specificity of 0.90 and 0.85, respectively.

It is of certain value to make the differential diagnosis of PA and AL by enhancing the maximum-level texture histogram analysis of CT images without extending the patient examination time and increasing the economic burden, and it can provide new ideas for clinical and imaging diagnosis. Limitations of this study: First, this study is retrospective, the sample size is small, there is a certain selectivity bias, and the sample size of the included studies between groups varies greatly, which may lead to error in the results. Although the sample size was small, we still found a strong association between texture features and the diagnosis of PA and AL, and we should proceed to investigate the association of broader radiological features with parotid gland tumors in a multicenter large sample. Second, although the ROI of tumor lesions is outlined with the participation of radiologists and oral and maxillofacial surgeons with extensive diagnostic experience, the selection of ROI is done manually, which leads to sampling bias. Third, this study only analyzes the first-order parameters of the most commonly used texture analysis method, histogram analysis, and the second-order parameters and high-order parameters have not yet been included in the study, and the later research will be further explored by algorithms such as auto-segmentation of radiomics and volume integration neural network.

## Conclusions

In conclusion, our findings indicate that enhanced CT combined with texture analysis provides more quantitative and reliable indicators for the differential diagnosis of PA and AL, which has certain value for the preoperative differential diagnosis of parotid gland tumors, and provides new ideas and methods for the further preoperative accurate differential diagnosis of parotid gland tumors in clinical work without increasing the economic burden of patients.

## Data Availability

Datasets used and/or analysed during the current study may be obtained from corresponding authors upon reasonable request.
